# Primary Urothelial Bladder Cancer in a Young Patient: A Case Report and Review of the Literature

**DOI:** 10.7759/cureus.15864

**Published:** 2021-06-23

**Authors:** Bandar Alhubaishy, Joachim Mathes, Thomas Knoll

**Affiliations:** 1 Urology, King Abdulaziz University Hospital, Jeddah, SAU; 2 Urology, Sindelfingen-Boeblingen Hospital, Sindelfingen, DEU

**Keywords:** hematuria, transurethral resection of the bladder tumor, urothelial bladder cancer, young patient, cystocopy

## Abstract

Despite being a rare condition among young patients, here, we report about a 22-year-old patient with primary urothelial bladder cancer. The patient complained of macroscopic painless hematuria. Transabdominal ultrasound revealed a 2-cm-sized exophytic lesion occupying the left-sided urinary bladder wall. The histologic examination of a specimen obtained during transurethral resection of the bladder tumor showed a superficial low-grade urothelial bladder tumor, pTa G1. Close follow-up with regular cystoscopies and urine cytological examinations is the cornerstone in the disease’s therapy. Underlying genetic factors may predispose to the development of the disease, which may require further investigations.

## Introduction

Bladder cancer represents the 10th most common cancer worldwide. Its incidence increases worldwide, particularly in developed countries [1,2]. It is more common in patients older than 55 years [3]. The incidence of bladder cancer is higher in male individuals than in female individuals. Smoking and occupational exposure to certain chemicals known as aromatic amines are considered the main risk factors. *Schistosoma haematobium* was the most common risk factor for bladder cancer in Egypt [4]. Hematuria is the most common presenting symptom and it requires further evaluation using transabdominal ultrasound, cystoscopy, and computed tomography [5]. Although several trials have failed to determine the main genetic key factors responsible for bladder cancer, several genetic loci have been discovered that may increase the susceptibility to develop bladder cancer [6]. Among the loci are MYC [7,8], NAT2 [9], and GSTM1 [10,11].

## Case presentation

**Figure 1:**
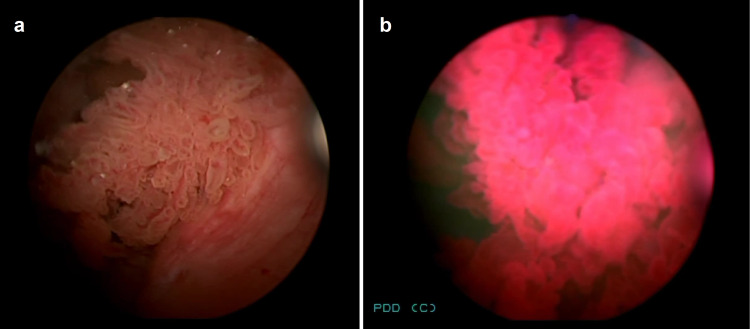
Cystoscopy on patient presentation showing a papillary bladder tumor: (a) in white light and (b) Hexvix-induced fluorescence of the bladder urothelial tumor in blue light.

**Figure 2:**
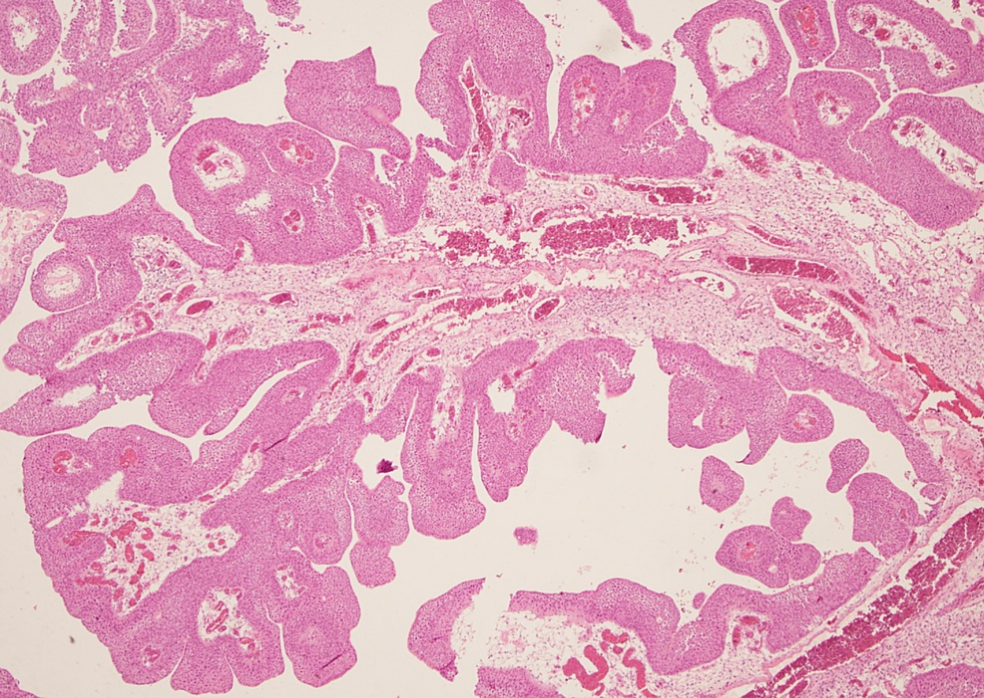
Histological finding of superficial low-grade urothelial bladder tumor, pTa G1

**Figure 3:**
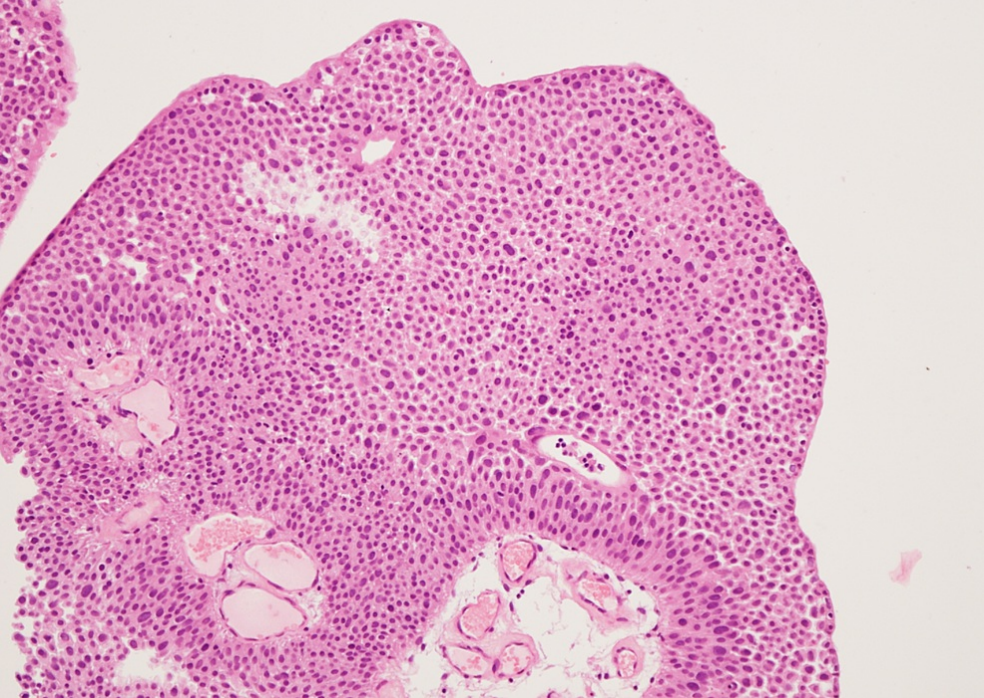
Histological finding of superficial low-grade urothelial bladder tumor, pTa G1

A 22-year-old man presented in January 2021 with a two-week history of total painless macrohematuria. He works in an office. Based on the patient’s medical history, there was no evidence of chemical product exposure or nicotine abuse. Family history of hereditary nonpolyposis colorectal cancer, Lynch syndrome, and other malignancies was denied. Physical examination showed no significant findings. Transabdominal ultrasound showed a mass occupying the left-sided urinary bladder wall. Consequently, a flexible white light cystoscopy in sedation was performed for further evaluation, and a 2-cm-sized papillary lesion occupying the left-sided urinary bladder wall was observed (Figure 1). The patient underwent transurethral resection of the bladder tumor (TURBT) and immediate post-TURBT mitomycin instillation. Histopathologic examination showed a superficial low-grade urothelial bladder tumor, pTa G1 (Figures 2, 3), with low risk of recurrence and progressions based on the European Association of Urology risk stratification system. Consequently, a computed tomography of the abdomen-pelvis and further genetic evaluations were not performed. Cystoscopic examination three months after the surgery showed no recurrence. The patient is under regular and intense follow-up through cystoscopy.

## Discussion

The present case is unique because of the patient’s age. Bladder cancer diagnosis and treatment in young patients are challenging because obligatory close follow-up that includes regular cystoscopies, urinary cytology tests, and pyelographies is recommended to evaluate the upper urinary tracts [12]. Several published articles have discussed behavioral and clinicopathologic patterns for this condition [12-16]. A retrospective study involving five patients aged 24 years (±2.83 years) examined the specific characteristics of bladder cancer [12]. The patients presented with painless hematuria. Three patients were diagnosed with pTa, and the remaining two patients had pT1 and pT2. One patient with pTa reported multiple recurrences and progressed to pT2. This patient declined radical cystectomy and received multimodality treatment. He died because of disease progression. The two remaining patients with pTa experienced no recurrence after the initial TURBT. The patient with pT1 experienced a single recurrence with a pTa. Subsequent follow-up for two years showed no evidence of recurrence. The last patient with pT2 underwent radical cystectomy and s-pouch diversion with the preservation of the genital organs. Urothelial tumors in young patients have distinguishable genetic characteristics compared with older patients based on a study that used a Lund subtype-specific immunohistochemistry panel to examine the molecular subtypes of a urothelial carcinoma among 49 patients aged <45 years [13] . The study revealed that 80% of patients had molecular urothelial-like A subtypes, which are characterized by improved recurrent-free survival. However, 10% of patients had molecular urothelial-like D type, which is characterized by high-grade non-muscle-invasive cancers with higher levels of squamous differentiation and p16, E2F3, and Ki-67 expression in addition to Ck20 expression and lower recurrent-free survival.

The clinical behavior of the disease was retrospectively reviewed in patients aged <40 years at a tertiary medical center in China from 1994 to 2004 [14]. These patients tended to have nonadvanced stages and low-grade disease and appeared to be less multifocal during initial presentation. Moreover, the young patients showed a lower recurrence than older patients. Further investigations regarding the clinicopathologic characteristics of urothelial bladder cancer patients, including 42 young patients (aged ≤30 years) and 2,783 older patients (aged >30 years), have been performed retrospectively. The investigations revealed that superficial low-grade and low-stage tumors were more common in young patients than in older patients, and improved prognosis was observed in young patients than in older patients [15]. A retrospective study compared 56 patients with urothelial bladder tumor between 2007 and 2013 who were aged <40 years to a control group of patients aged >40 years during the same period with respect to clinical behavior, pathologic characteristics, and prognosis [16]. This study showed that the younger bladder cancer patients tended to have low-grade smaller-sized tumors (<3 cm) and higher papillary urothelial neoplasms of low malignant potential than patients aged >40 years. The five-year survival rates of young and old patients were 100% and 88.1%. respectively. Regarding the recurrence-free and progression-free survival rates, no difference was observed between the two groups.

## Conclusions

The management of primary urothelial bladder cancer in young patients is challenging because of the necessity for close follow-up. Although a majority of younger patients have low-grade noninvasive urothelial tumors and favorable prognosis compared with older patients, a small percentage may manifest with high-grade invasive tumors that have poor prognosis. Genetic etiologies may also contribute to the pathogenesis of this disease in this patient group.
